# Controlled Semantic Cognition: Precision Recordings Converge With in Silico Experiments to Reveal the Inner Workings of the Anterior Temporal Lobe Hub

**DOI:** 10.1162/NOL.a.220

**Published:** 2026-03-17

**Authors:** Rebecca L. Jackson, Guy A. Orban, Paul Tiesinga

**Affiliations:** Department of Psychology, University of York, York, UK; York Biomedical Research Institute, University of York, York, UK; Department of Medicine and Surgery, University of Parma, Parma, Italy; Donders Institute for Brain, Cognition and Behaviour, Radboud University, Nijmegen, The Netherlands

**Keywords:** computational modelling, functional connectivity, Granger causality, intracortical EEG, semantic cognition, semantic control

## Abstract

The anterior temporal lobe (ATL) is crucial for learning and storing concepts, yet the inner workings of its multiple cytoarchitectonically distinct subregions remain a “black box.” Moreover, it is not yet clear how this region interacts with a distributed network of brain regions to access context-specific information. However, two recent papers have made crucial steps forward, while adopting radically different approaches. One (Jackson et al., 2021) used in silico experimentation to ask how a system can meet the core requirements of controlled semantic cognition, comparing multiple theoretical architectures on their ability to meet these demands. The other (Tiesinga et al., 2023) performed in vivo intracortical recordings with unprecedented coverage and spatial precision, comparing cytoarchitectonic ATL subregions. In this perspective, we bridge these recent in silico and in vivo explorations of controlled semantic cognition, demonstrating their remarkable convergence. Thereby, we expose the inner workings of the ATL semantic hub and the regulating effect of frontal regions, while highlighting the crucial next challenges. The result is a neuroanatomically precise mechanistic model of controlled semantic cognition.

## INTRODUCTION

Semantic cognition, the acquisition and controlled use of multimodal conceptual knowledge, underpins our everyday interactions with people and our environment (Lambon Ralph et al., 2017; Patterson et al., 2007). The brain combines information across multiple senses, moving beyond superficial perceptual similarities to uncover the conceptual structure necessary to drive meaningful behaviour (Rogers & McClelland, 2004). For example, using shape information in isolation, a pear would appear more similar to a lightbulb than an orange, resulting in an inappropriate behavioural response. To respond similarly to different fruits, we must also factor in somatosensory, gustatory, and olfactory features. In semantic dementia (also referred to as semantic variant primary progressive aphasia; Gorno-Tempini et al., 2011), these multimodal semantic representations are gradually lost as the bilateral anterior temporal lobes (ATLs) progressively deteriorate (Patterson et al., 2007; Snowden et al., 1989; Warrington, 1975). This results in a profound impairment of verbal and nonverbal comprehension as patients lose their ability to communicate (e.g., not being able to name an orange) and interact in an informed fashion with their environment (e.g., knowing to peel the orange before eating it). Thus, the bilateral ATL was identified as the area responsible for bringing together information acquired in different senses. As a direct consequence of learning to map between sensory-specific “spoke” areas, multimodal concept representations emerge in this “hub” region (Lambon Ralph et al., 2017; Patterson et al., 2007; Rogers et al., 2004). This is known as the hub-and-spoke theory of semantics. Subsequently, a broad range of neuroimaging and neurostimulation approaches have confirmed the importance of the ATL in multimodal semantic representation (Abel et al., 2015; Binney et al., 2012; Chen et al., 2016; Devlin et al., 2000; Jackson et al., 2016; Marinkovic et al., 2003; Pobric et al., 2007; Shimotake et al., 2015; Visser, Jefferies, & Lambon Ralph, 2010; Visser & Lambon Ralph, 2011). The relative engagement of the left and right ATL varies somewhat based on the modality of input (e.g., written words elicit more left ATL activity; Rice et al., 2015) and features of the concept (e.g., social concepts often elicit more right ATL activity; Olson et al., 2007; Rice et al., 2015; Zahn et al., 2007). However, both are engaged in semantic judgements across all concepts (Abel et al., 2014; Platonov et al., 2019; Rice et al., 2015, 2018), their connectivity is crucial (Jung & Lambon Ralph, 2016, 2023), and only bilateral damage has severe consequences (Lambon Ralph et al., 2010, 2012). They may therefore be considered two parts of one system which work in concert to represent multimodal semantic concepts (Rice et al., 2018). Although several models have been proposed for the semantic system and much debate remains, the evidence increasingly supports both the importance of spokes (Fernandino et al., 2016; Meteyard et al., 2012; Pulvermüller, 2013) and the critical role of the ATL hub, which is now well accepted (Binder et al., 2011; Lambon Ralph et al., 2017; Kuhnke et al., 2023; Fernandino & Binder, 2024). Additional brain areas respond to multimodal semantic concepts, yet damage to these regions does not result in the loss of semantic representations, suggesting they fulfill distinct functional roles (Jefferies & Lambon Ralph, 2006; Humphreys & Lambon Ralph, 2015; Humphreys et al., 2022). While the hub-and-spoke regions extract and represent stable concepts, additional regions are required to select the conceptual information relevant to a particular context or task (Davey et al., 2015; Jackson, 2021; Jefferies, 2013; Noonan et al., 2013; Reilly et al., 2025). These *semantic control* processes are underpinned by a left-lateralised network focused on inferior frontal and posterior temporal cortices (Davey et al., 2015; Jackson, 2021; Jefferies, 2013; Jefferies & Lambon Ralph, 2006; Noonan et al., 2013; Thompson et al., 2015).

The brain learns multimodal semantic concepts as a consequence of mapping between features in different sensory modalities. Artificial neural networks (ANNs) also uncover relationships in their (training) environment by learning to map between features. Consequently these models can simulate how semantic concepts are learnt. Just like the cortical semantic system, ANNs can extract the higher order structure of their environment as they learn to map from provided (input) to required (output) features. In a model of the semantic system, these input and output features may be presented to discrete regions (layers) reflecting different (real or artificial) senses. This model could then be assessed on its ability to accurately learn the multimodal conceptual structure necessary for semantically-driven behaviour (e.g., representing pears similarly to oranges) based on the relationships between features experienced across multiple sensory modalities and events (Cao et al., 2020; Jackson et al., 2021; Saxe et al., 2019). This ANN could have a wide range of architectures (configurations of units and their connections) that may be (a) better or worse at uncovering this underlying conceptual structure and (b) further from, or closer to, the way the brain solves this difficult problem. As such, constructing a single model may not accurately demonstrate how the cortical semantic system works. A powerful alternative is to systematically manipulate architectural features across multiple ANNs and measure the impact on performance. One can then examine whether this reverse-engineered solution utilises a similar architecture to the cortical system.

## REVERSE ENGINEERING THE SEMANTIC NETWORK

**Figure 1.**
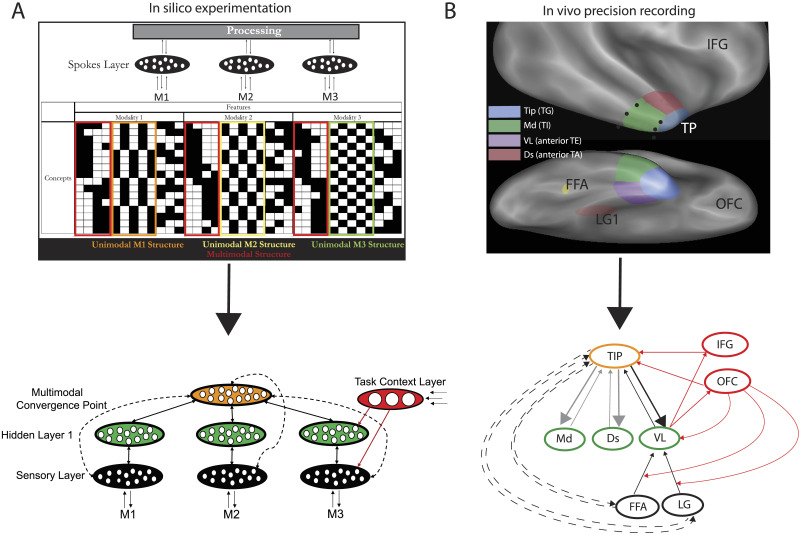
The convergence between precision stereo electroencephalographic (sEEG) recordings of the cortex (from Tiesinga et al., 2023) and a model reverse engineered to promote controlled semantic cognition (from Jackson et al., 2021). (A) In silico experimentation compared a series of architectures with this basic setup on their ability to learn the underlying conceptual structure while responding with the task-appropriate subset of features. Top: The grid shows the features of each concept in the model environment (black features are present for that concept). The environment included features in three different artificial sensory modalities, each of which had its own structure, as well as features related to a multimodal structure (shown by the coloured boxes). The architectures varied on their connections and the presence of one or two hidden layers but all had sensory regions to allow the input and output of features in each sensory modality and the same number of units and connections overall. The models received features of a concept in one of the three modalities, and a task-context signal designating the modality in which output was required, resulting in nine different possible tasks (e.g., experiencing a concept’s features in modality 2 and responding with its features in modality 3). Bottom: The optimal model architecture identified by Jackson et al. Black = sensory input and output regions in each of the three modalities; green = hidden units for processing in a shallow hidden layer connected to the sensory areas; orange = hidden units for processing that connect the different processing streams resulting in multimodal convergence; red = control regions providing the task context. (B) The temporal pole (TP) subregions explored with sEEG and their proposed correspondence with the model layers. Top: The four TP subregions are shown on lateral and ventral views of the inflated MNI brain. Blue = tip; green = ventrolateral (VL); red = dorsal; and purple = medial subregions. The black dots indicate the positions used to calculate the approximate *y* coordinate of the rostral and caudal borders of VL used in Figure 2. The yellow and red hatching indicate the FFA and lingual gyrus regions providing visual input to TP. The labelled frontal regions correspond to the regions included in the network figure below as red ellipses. FFA = fusiform face area; LG = lingual gyrus; IFG = inferior frontal gyrus; OFC = orbitofrontal cortex. Bottom: Functional connections between tip, VL, and ventral PTC (vPTC) regions (FFA and lingual gyrus), OFC, and rostral IFG found using Granger causality in Tiesinga et al. This utilises the same colour code as the model in (A). Connections identified with putative control regions are coloured red. The shortcut connections are displayed with dashed lines. Black (and red) lines represent connections identified in Tiesinga et al., while grey lines show connections that are assumed to be important for nonvisual processing based on prior work and that showed weaker involvement in Tiesinga et al. These connections link tip with the dorsal (D) region (corresponding to anterior TA), processing auditory and also somatosensory information, and within the medial (Md) subregion (specifically with TI), processing olfactory information (Ding et al., 2009).

In recent work, Jackson and colleagues (2021), adopted this reverse-engineering approach to determine how a semantic system should be organised. Recurrent ANNs (ANNs with both feedforward and feedback connections) were constructed with seven different architectures, reflecting different theoretical perspectives on the cortical semantic system (Binder & Desai, 2011; Damasio et al., 1996; Eggert, 1977; Lambon Ralph et al., 2017; Martin & Chao, 2001; Patterson et al., 2007). This included simulations with unimodal processing regions only, with a multimodal hub bridging all sensory modalities, or with multiple bimodal hubs bridging between pairs of modalities (or a combination of these). In addition, the architectures varied in depth (whether the processing units formed one or two layers) and the presence of “shortcut” connections: a small number of direct connections (in either direction) between the sensory input/output areas and the multimodal hub, which therefore skip the intervening layer. While conceptually similar to skip connections in deep ANNs (He et al., 2016), these shortcut connections do not project all information contained in one layer to a later layer, but simply connect pairs of units via learned weights (i.e., they are the same as any other connection in the model). They may therefore have greater biological plausibility as well as different functional consequences. All models had an equal number of resources (units and weights) and were trained until they could successfully associate the features of each concept across all possible combinations of three artificial sensory modalities, resulting in nine different tasks (Figure 1A, top). After learning, the models were compared on how well they identified the full multimodal conceptual structure present in their environment, utilising all sensory features presented across multiple modalities and task contexts and not merely those active in the current task context. This is comparable to asking whether presenting a picture of a pear results in a similar activity pattern to an orange, and not just a lightbulb, using representational similarity analysis (Kriegeskorte & Kievit, 2013; Kriegeskorte et al., 2008).

These in silico experiments highlighted three architectural features as beneficial: the presence of a single multimodal hub, a deep network, and shortcut connections. The “winning” architecture is shown in Figure 1A. Both Jackson et al. (2021) and Rogers et al. (2004) demonstrate that a single area responsible for mapping between the information in each sensory area forms multimodal concept representations, whilst separate connections between the various sensory regions cannot. Moreover, Jackson et al. demonstrated that this requirement for one multimodal representation area cannot be solved by different areas combining information from each pair of senses. Indeed, these bimodal regions cannot even be present in combination with the multimodal hub, as this obviates the need to utilise it for each mapping. This mechanistic account demonstrates why both unimodal sensory areas and a single multimodal hub, located in the ATL, are required for healthy semantic cognition. Additionally, Jackson et al. extended the hub and spoke theory by demonstrating the importance of depth (also see Chen et al., 2017), which allows the system to perform multiple, sequential transformations on the input, allowing different layers to represent information at different levels of abstraction. This means shallow layers can extract the structure within a sensory modality and deeper layers can identify relationships across modalities. Furthermore, Jackson et al. highlighted the dramatic effects of a small number of direct, shortcut connections between sensory areas and the multimodal hub, a novel addition to the hub and spoke theory.

Jackson et al. (2021) also addressed the critical question of how the two crucial semantic processes, control and representation, coexist given their antagonistic demands. Semantic representation, the acquisition and storage of conceptual knowledge, is not sufficient for successful semantic cognition. The need for flexible context-appropriate behaviour, combined with the vast amount of information stored about any particular concept, necessitates additional control processes (Jefferies, 2013; Lambon Ralph et al., 2017). This semantic control inhibits irrelevant, yet often prepotent associations, allowing only selected task-relevant features to drive behaviour. For example, while it would be unwise to treat them similarly when seeking a food source, if given instructions to sort by shape, one could ignore the strong association with the orange to correctly match the pear and lightbulb. This requirement poses a challenge for the semantic system. On the one hand, it must combine features from different sensory modalities and different encounters to extract the underlying multimodal conceptual structure. On the other hand, it must act only upon the features relevant to a particular situation by shaping activity in the semantic network based on a goal or task context. For the first time, Jackson et al. simulated the inhibition and selection of semantic features in a recurrent framework, by limiting the subset of features allowed in the response. Instead of activating all features of a concept, different types of features were retrieved in different tasks. They demonstrated that a semantic system can support both the interacting, yet antagonistic, task context-independent multimodal representation and context-dependent control processes, if distinct areas develop a relative functional specialisation for each process. Architectures that represented task-context and task-invariant conceptual information in distinct areas were able to selectively respond with features in the required domain, whilst successfully extracting multimodal conceptual representations (see Figure 1A).

Jackson et al. (2021) chose their optimal model architecture solely on the quality of the conceptual information extracted during learning, without considering similarity to the neural system. Despite this, it displays remarkable agreement with the coarse structure of the cortical semantic network. There is strong evidence for the presence of a single multimodal hub in the ATL with damage to this region alone resulting in a loss of multimodal semantic representations (Patterson et al., 2007; Snowden et al., 1989; Warrington, 1975). Similarly, there is a clear need for multiple layers to support the transformation from sensory to semantic representations, as demonstrated by the correspondence between differing layers of deep ANNs and regions along the ventral visual stream (Kriegeskorte, 2015; Yamins & DiCarlo, 2016; also see Hauk et al., 2024). Additionally, the cortex displays a relative functional specialisation for semantic control and representation (Jackson et al., 2021; Jefferies, 2013; Jefferies & Lambon Ralph, 2006; Lambon Ralph et al., 2017). While the ATL supports semantic representation, lateral frontal cortex and posterior temporal cortex (PTC) underpin semantic control (Jackson et al., 2021; Jefferies, 2013; Noonan et al., 2013), with damage to either control area resulting in inconsistent semantic access across task contexts, relying heavily on prepotent associations (Jefferies & Lambon Ralph, 2006; Thompson et al., 2015). Critically, this correspondence suggests many of the architectural features identified in the reverse-engineering process are also utilised by the brain to meet the difficult requirements of a semantic system, and therefore the reverse-engineered model can serve as a mechanistic account of the neuronal semantic network. However, for some of the predicted features, there is currently only limited evidence that they are implemented within the cortex. Here, we highlight the results of a recent study by Tiesinga et al. (2023; see Figure 1B) which begins to address the remaining hypotheses, supporting the reverse-engineered model by further demonstrating its close alignment with the cortical semantic system (Figure 1) and highlighting crucial areas for further exploration.

## SPATIOTEMPORALLY PRECISE INTRACORTICAL MEASUREMENT WITHIN THE ATL HUB

Tiesinga et al. (2023) recorded from stereo electroencephalography (sEEG) leads implanted for diagnostic purposes in the most anterior aspect of the ATL, the temporal pole (TP), and the neighbouring cortex of epileptic patients (Figure 1B). Patients watched short (1,167 ms) videos showing a male or female actor performing one of two object manipulations (dragging or grasping), preceded by a static presentation of the first frame (for 275 or 875 ms). Tiesinga et al. employed a factorial design with two tasks—a semantic gender discrimination and a nonsemantic action discrimination—which were performed equally well (97% correct; Platonov et al., 2019). “Male humans” and “female humans” are multimodal semantic concepts, and discriminating them required, according to Tiesinga et al., the combination of visual information about the face and hand, which are known to remain segregated even at the highest levels of the visual system (Premereur et al., 2016). On the other hand, discriminating basic actions is independent of the semantic system, instead depending upon praxis systems within parietal cortex (Aflalo et al., 2020; Orban et al., 2019), as demonstrated by neuropsychological double dissociations (Adlam et al., 2013; Corbett et al., 2009; Kalénine et al., 2010) and neuroimaging (e.g., Culham & Valyear, 2006; Urgen & Orban, 2021). Note, that here the task is a simple discrimination between two actions that does not utilise action events which may also recruit the ATL (Binder & Desai, 2011; Desai et al., 2023; Fernandino et al., 2013). Analyses were limited to the broad gamma frequency band as this reflects neuronal activity (Burke et al., 2015; Manning et al., 2009; Ray et al., 2008; Ray & Maunsell, 2011; Xie et al., 2024). An earlier sEEG study utilising these videos and tasks revealed responses restricted to TP and orbitofrontal cortex (OFC) bilaterally but with a clear right hemisphere bias (Platonov et al., 2019). Therefore, Tiesinga et al.’s analyses were restricted to the right hemisphere. In both studies, TP responses were brief (< 150 ms), independent of stimulus duration, and task-dependent. Tiesinga et al. first investigated how the semantic task responses differed across subregions of the right TP.

**Figure 2.**
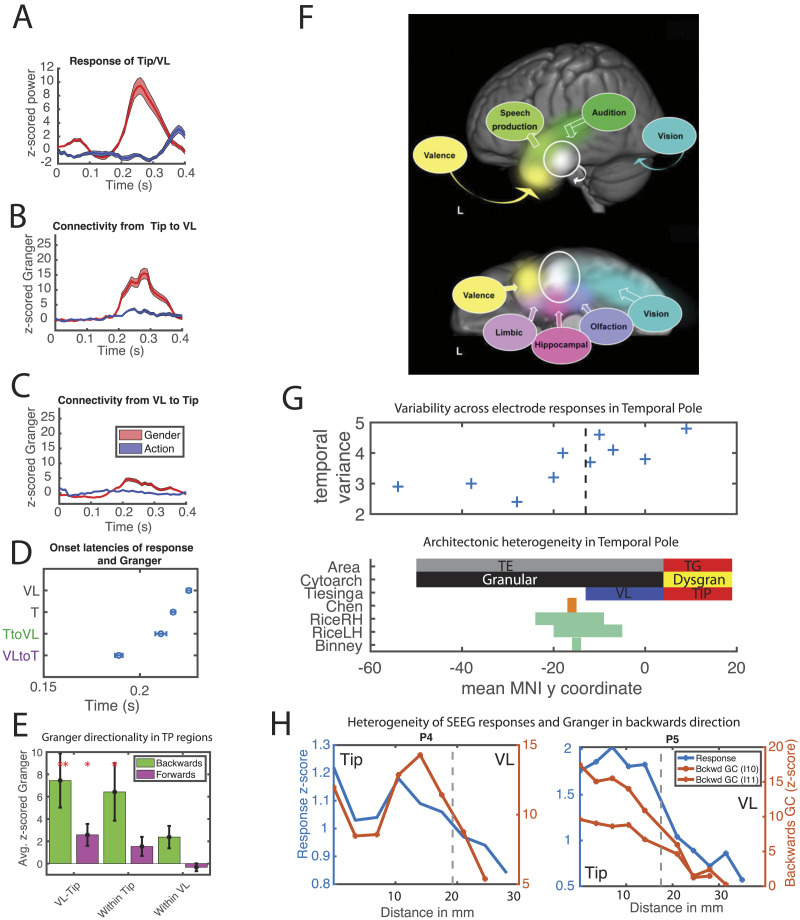
Key findings from Tiesinga et al. ’s (2023) sEEG recordings and their relation to prior sEEG and fMRI studies. (A–D) The sEEG data of one patient (see T23 for data from 4 patients). (A) Time courses of the responses in tip and VL. (B) Connectivity (Granger causality [GC] at the short delay) backward from tip to VL. (C) The connectivity (GC at short delay) forward from VL to tip in the semantic and nonsemantic tasks. (D) Onset of these responses and connections (at the short delay). In each patient, the connectivity from VL to tip preceded the connectivity from tip to VL and the tip and VL responses. (E) The group average strength of the forward (purple) and backward (green) connections within and between tip and VL for the semantic task. (F) The graded hub-and-spokes scheme from Rice et al. (2015) shown on the lateral and ventral views of the left hemisphere, including a basal multimodal convergence point (shown in white) corresponding approximately to VL. (G) Locating anterior temporal lobe (ATL) structure and activity on the rostrocaudal dimension. The top diagram shows each electrode in Rogers et al. (2021) plotted at its location on the *y* axis in MNI space by the amount of variance in the coefficient change for the classifier fitted to the raw sEEG signal (adapted from Rogers et al., 2021), reflecting the changing nature of the representation at that location over time. This is stronger for more anterior electrodes, and strongest for the one electrode judged to be within tip (see scale in the lower part of the diagram). The lower diagram utilises the same scale and displays the location of areas TG and TE and their cytoarchitectural differences, as well as tip and VL as defined by Tiesinga et al. (2023; corresponding to TG and anterior TE, respectively). TP extends from the rostral end of the temporal pole to the limen insulae, as in Ding et al. (2009) and Pascual et al. (2015). Additionally, the locations of local maxima in prior studies are shown, including that from the earlier ECoG study by Chen et al. (2016; brown) and fMRI studies (green). This includes the range at which right and left hemisphere peaks were identified in the multiple fMRI studies presented in Rice et al. (2018; Table 1); and the peak fMRI location from Binney et al. (2010), which has been repeatedly used as a vATL region of interest (ROI; ±36, −15, −30). (H) For the two electrodes in two patients which included both tip and VL regions in Tiesinga et al., response strength and feedback connectivity (towards posterior TP) are plotted as a function of distance from the most rostral lead of the electrode, inserted almost perfectly parallel to the *y* axis. The grey dashed line shows the division between VL and tip. More anterior locations showed stronger feedback connections and stronger responses, resulting in the overall differences between tip and VL.

Tiesinga et al. (2023) distinguished four TP subregions based on the cytoarchitectonic regions defined in Ding et al. (2009) and their differences in functional connectivity (Pascual et al., 2015; see Figure 1B). The most anterior region (cytoarchitectonic TG) was labeled *tip*. Posterior to this, the TP was separated into dorsal (anterior TA), ventrolateral (VL; anterior TE), and medial (TI, 35, 36; entorhinal cortex) subregions (see Figure 2G). These four subregions have differing functional connectivity, focused on auditory and somatosensory regions, default mode network and local paralimbic regions, respectively. Tip and VL were separated despite having similar default mode network functional connectivity (Pascual et al., 2015), as they critically differ in cytoarchitectonics (dysgranular vs. granular), chemoarchitecture (density of SMI 32 [nonphosphorylated neurofilament protein] and WFA [wisteria floribunda agglutinin] labelling; Ding et al., 2009), structural connectivity (Sasaki et al., 2023), and intra-TP and subcortical functional connectivity (Pascual et al., 2015). In contrast, areas grouped within the medial subregion share chemoarchitectural and cytoarchitectonic features (Ding et al., 2009). Semantic task responses were focused in tip and, to a lesser extent, VL.

**Figure 3.**
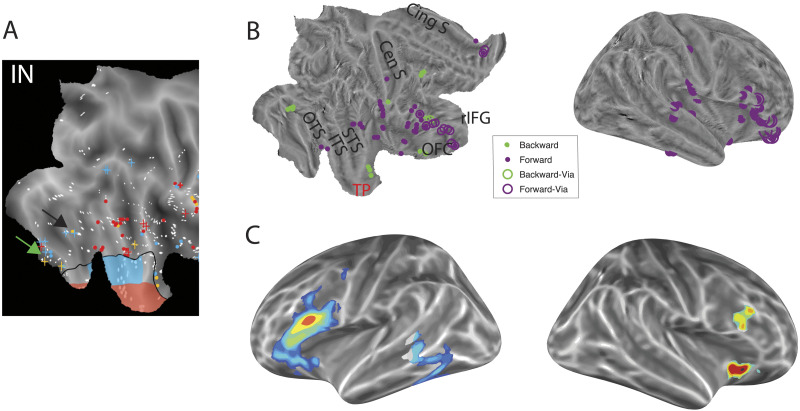
The locations of TP inputs and areas implicated in semantic control. (A) The location of leads providing input (IN) to VL (blue), tip (red) or both (yellow) over a short (dots) or long (crosses) delay, as determined by Tiesinga et al. (2023) using GC. Green and black arrows point to lingual gyrus and FFA, respectively. (B) The location of leads conditioning the short delay GC from VL to tip (purple), shown on both a flatmap and an inflated right hemisphere (from Tiesinga et al.). Via leads (open circles) indicate conditioning leads that also have established connections with each region. OTS = occipitotemporal sulcus; ITS = inferior temporal sulcus; STS = superior temporal sulcus; OFC = orbitofrontal cortex; rIFG = rostral inferior frontal gyrus; Cen S = Central sulcus; Cing S = cingulate sulcus. (C) The areas consistently implicated in semantic control across both hemispheres from an activation likelihood estimation meta-analysis of neuroimaging studies (Jackson, 2021) projected onto the cortical surface of left and right hemispheres for comparison with (B).

Tiesinga et al. (2023) then used Granger causality (GC) to trace the route and direction of information flow between pairs of leads within TP and between TP and other right hemisphere areas (Box 1). Granger causality allows the measurement of directional information flow (Chapeton et al., 2022). GC was assessed at a short (4 ms) and a long (16 ms) delay, covering the range of neuronal interareal connections (0–20 ms; Banaie Boroujeni & Womelsdorf, 2023; van Kerkoerle et al., 2014). As expected, connections with a short delay were more frequent between TP subregions than with non-TP areas. Remarkably, the task-dependent functional connections were as short-lived as the TP responses (Figure 2A–C). Within the TP, information travelled first from VL to tip and then back again (Figure 2B–D). Intriguingly, these backward connections were stronger than their forward counterparts (Figure 2E), and more frequently direct (assessed using conditional GC; see Box 1 and also Figure 3B). Additionally, Tiesinga et al. identified regions providing input to, or receiving output from, the TP, highlighting the dynamic connectivity with prefrontal cortex and PTC. The time courses of the TP responses were highly related to the timing of their incoming connections (more than the timing of activity in their input regions). This intracortical assessment provided a crucial window into the time-resolved activity and connectivity of ATL subregions during a semantic task, with high spatiotemporal precision and coverage across the TP.

**Box 1.** Granger causality analyses characterise the directed connectivity within the semantic network. sEEG recordings summarise the synaptic input and spiking activity over approximately 1 cubic mm around each contact point (channel; Mercier et al., 2022). The dominance of broad gamma band power in the response periods indicated that Tiesinga et al.’s (2023) measurements did reflect the spiking activity of neurons, at least in part (Leonard et al., 2024; Xie et al., 2024). Unlike coherence measures (Anand et al., 2023), Granger causality (GC) analyses allow inference of the direction of interaction between two electrode channels. To that end, GC quantifies the extent to which predictions of the activity in one channel are improved by taking into account the previous activity on the other channel, over and above its own history. Tiesinga et al. achieved this by fitting an autoregressive (AR) model to the channel activity. While GC is designed for stationary signals, the nonstationary dynamics of the interactions could be assessed by applying GC to short (100 sample) windows that could be considered approximately stationary, which are then shifted across the trial interval in order to obtain a time-varying GC. The number of model parameters, which is the product of the square of the number of channels considered and the number of previous samples included in the AR model, needs to be balanced with the amount of data available in the window. For the analysis presented in Tiesinga et al., this amounted to four prior samples for a pair of channels. Taking samples every 1 or 4 ms resulted in maximum delays of 4 and 16 ms, covering the appropriate delays for probing direct communication between neighbouring and distal regions (Banaie Boroujeni & Womelsdorf, 2023; van Kerkoerle et al., 2014), respectively. As some interactions may be task-independent, the GC measure was *z* scored relative to the baseline prior to the onset of the static image. While standard GC analyses are directional, they fall short of identifying direct causal influences as a common input affecting the two channels could lead to the detection of spurious connections. This can be addressed by conditioned GC, where the predictive model includes additional channels to factor out such common input. Using this procedure it was possible to distinguish direct connections from indirect interactions mediated by a third region. Taken together, *z*-scored conditioned and unconditioned GC are a powerful tool to dissect the flow of information in a spatiotemporally precise way, revealing the dynamic interactions across a hitherto unappreciated regional heterogeneity in the temporal lobe.

Tiesinga et al. (2023) noted considerable agreement between their findings and the reverse-engineered model of Jackson et al. (2021). Note that while the formal model only contains three layers, this is a simplification; increasing the depth was beneficial and the cortical system requires many steps to go from sensory input to conceptual knowledge (Kriegeskorte, 2015; Yamins & DiCarlo, 2016). Thus, the three model layers may be mapped to the many cortical layers at various different spatial scales, representing more or less of the perceptual–conceptual system. Crucially, the deepest layer must correspond to the location where different sensory streams converge. The neuroimaging simulations in Jackson et al. represent a relatively large scale, with the input layer reflecting higher order sensory information in occipitotemporal cortices and the deepest multimodal layer reflecting the entire ATL. Here the focus is on a more granular scale: an in-depth look at the computations within the temporal lobe, starting with high-level sensory features. Specifically, Tiesinga et al. mapped PTC to the sensory input layer, VL to the shallower processing layer (with unimodal visual feedforward input) and tip to the deepest processing layer (where the feedforward input from multiple modalities converges; see Figure 1B, bottom). This is an appropriate spatial resolution for comparison with intracortical recordings that allows us to address novel questions regarding fine scale TP neuroanatomy. Here, we highlight the strong convergence between the reverse-engineered model and the intracortical data, providing further evidence for the similarity of the model architecture to the brain and adding neuroanatomical detail (Figure 1). We consider the wider implications of the three main points of convergence in turn: the functional organisation of the multimodal ATL hub, the importance of feedback and shortcut connections, and the interaction between semantic control and representation areas.

## FUNCTIONAL ORGANISATION OF THE ATL MULTIMODAL HUB

The findings reported by Tiesinga et al. (2023) are in strong accord with earlier intracerebral electrode studies demonstrating evidence for a multimodal hub in the ATL (Chen et al., 2016; Cox et al., 2024; Rogers et al., 2021; Shimotake et al., 2015). However, the ATL is large and inhomogeneous, consisting of multiple cytoarchitectural regions (Ding et al., 2009; see also Figure 1B). Where precisely do the multiple sensory processing streams converge in the ATL? This *multimodal convergence point* corresponds to the deepest layer of the reverse-engineered model and accurate identification of this locale is crucial to comprehend the wider organisation of the ATL. Indeed, Jackson et al. (2021) demonstrated the emergence of a graded hub centred on this region; conceptual representations are maximally multimodal here and, while all prior layers have bottom-up input in a single sensory modality, their representations are increasingly multimodal as they get closer to this area due to its top-down feedback. This aligns with the cortical evidence demonstrating a gradual transition from largely unimodal to highly multimodal involvement across temporal cortex (Bajada et al., 2017; Cox et al., 2024; Jackson et al., 2018; Lambon Ralph et al., 2017; Rice et al., 2018). However, questions remain regarding the precise location of the multimodal convergence point. Tiesinga et al. may better separate the role of ATL subregions than earlier ECoG studies as their direct sEEG recordings of the grey matter have three critical advantages: (1) coverage of the entire cortical surface rather than only the relatively flat cortical gyri; (2) better spatial resolution, with leads separated by 3.5 mm rather than 2 cm; and (3) more anterior coverage of the temporal lobe, extending into the tip of the TP. Note, that while Abel et al. (2014, 2015) used a specialised ECoG array to extend their coverage anteriorly, their spatial resolution remained limited with only a few electrodes in tip.

Tiesinga et al. (2023) demonstrated the importance of distinguishing TP subregions, as both tip and VL had more semantic task-responsive electrodes than the medial and dorsal subregions. Critically, this aligns with prior ECoG and fMRI studies highlighting the basal anterior temporal surface, focused around VL, as the key site for multimodal conceptual representation (Chen et al., 2016; Rogers et al., 2021; Shimotake et al., 2015). This basal area (described as between 3.8 and 5.2 cm from the pole within anterior fusiform gyrus) is active and necessary across modalities and tasks (Shimotake et al., 2015) and was demonstrated to encode semantic content using a searchlight representational similarity analysis (Chen et al., 2016; Cox et al., 2024). Tiesinga et al. provided additional supporting evidence for the importance of this basal region. During the semantic task, VL received information from both fusiform and lingual gyri and had a high number of task-responsive leads. Moreover, the latency of VL responses correlated with reaction times in this task. However, the prior ECoG assessments specifically identify this ventral area as the critical ATL region for multimodal conceptual processing, while Tiesinga et al. also highlighted the key role of tip. Tip had the most task-responsive leads and provided the majority of the TP outputs. Furthermore, the GC analyses indicated that information first flows from VL to tip, and only later travels in the opposite direction (Figure 2B–D). This pattern of receiving early input from VL is more consistent with tip being the deepest processing layer where the different modalities converge, rather than VL, as the input must first travel from sensory areas to the multimodal convergence point before this region feeds back to influence processing within the different sensory streams (Figure 2F). We could instead assume that VL is the multimodal convergence point and tip sits atop a sensory pathway, for example, by providing emotion information from OFC when viewing faces (i.e., the valence pathway postulated by Rice et al., 2015). However, if this was the main sensory pathway driving VL activity one would expect the initial influence to be from tip to VL, and not vice versa. Additionally, if tip was responsible for valence, we would expect a greater impact of OFC on tip than VL activity, yet the direct effects on the two regions were equivalent. Hence, parsimoniously, Tiesinga et al. inferred an alignment between the cortical data such that tip is the likely locus of the multimodal convergence point, corresponding to the deepest layer of the Jackson et al. (2021) model, while VL corresponds to the intermediate layer between the multimodal tip and the visual lingual and fusiform gyri (see Figure 1B, bottom). As such, all of VL’s bottom-up input would be expected to be unimodally visual, yet it would be hypothesised to show multimodal responses due to the strong feedback from the proximal tip region.

Tiesinga et al. (2023) proposed that the information represented in tip consists solely of a label that binds together all the features of a specific multimodal concept (such as female), which are represented within shallower regions, including VL. This identifier should not be confused with a verbal label, such as the word “male.” Note that Tiesinga et al. used a visual task only and inferred that tip likely has multimodal representations because its responses and connectivity identified it as the deepest part of the processing stream for the multimodal concepts, male and female. Direct tests of the convergence of information from multiple different senses have indicated the multimodal nature of VL (Shimotake et al., 2015) and, to some extent, tip (Abel et al., 2015; Shimotake et al., 2015). Systematically assessing the convergence across the ATL of information presented in different sensory modalities remains a critical goal for future research, due to the unimodal nature of Tiesinga et al.’s tasks and the fallibility of Granger causality estimates. However, bridging the computational and intracortical investigations has provided testable hypotheses for these crucial future investigations: that the tip would indeed respond to stimuli from various presentation modalities and that the relative difference in activation across modalities would be somewhat larger in VL than tip.

By comparing their sEEG results to the computational model, Tiesinga et al. (2023) identified tip (cytoarchitectonic region TG) as the most likely hypothetical location of the multimodal convergence point, while prior ECoG studies have focused on VL (cytoarchitectonic anterior TE). In either case, VL would demonstrate multimodal responses when tested directly, due to the impact of conceptual information fed backwards from tip. Thus, the only discrepancy of Tiesinga et al.’s proposal with prior intracortical studies is that these did not explicitly identify tip. Because only the most posterior edge of tip was assessed in prior ECoG studies and these most anterior electrodes were typically heavily implicated (Chen et al., 2016; Rogers et al., 2021; Shimotake et al., 2015), the focus on VL may simply result from the extremely limited tip coverage in these earlier studies (see Figure 2G). Consistent with a multimodal role, tip demonstrates resting-state functional connectivity with all other TP subregions (Pascual et al., 2015), while VL is functionally connected to the pulvinar (Pascual et al., 2015; Zhou et al., 2016), a property typical of higher order visual areas. Furthermore, the strong dominance of temporal cortex structural connections in the anterior–posterior over the dorsal–ventral direction (Binney et al., 2010; Ding et al., 2009; Herbet et al., 2018; Jung & Lambon Ralph, 2023; Morán et al., 1987) appears more compatible with processing streams converging at the anterior tip of the TP and not on the basal surface. However, fMRI assessments demonstrate peak semantic activation in a ventral ATL region overlapping VL and not tip (see Figure 2G), supporting the prior intracortical studies’ focus on VL. These fMRI peaks are consistently posterior to the caudal border of tip, which we estimate to be located at a *y* coordinate of approximately +4 in MNI space (by averaging the 3 points on the boundary shown in Figure 1B, top). Eight fMRI studies performed between 2000 and 2015 (Binney et al., 2010; Devlin et al., 2000; Hoffman et al., 2015; Jackson et al., 2015; Sharp et al., 2004; Visser & Lambon Ralph, 2011; Visser, Embleton, et al., 2010, Visser et al., 2012) yielded local maxima in VL with a *y* coordinate ranging from −24 to −5, all at least 9 mm behind the estimated caudal border of tip (Figure 2G). Indeed, the peak coordinate from Binney et al. (2010), which has frequently been used to define a ventral ATL ROI (Gore et al., 2022; Jackson et al., 2016; Jung & Lambon Ralph, 2023; Rice et al., 2018), is located at (−36, −15, −30), approximately 20 mm posterior to tip. Several explanations may account for the discrepancy between the sEEG analyses and these fMRI peak coordinates in locating the likely multimodal convergence point within the ATL.

Firstly, Tiesinga et al. (2023) could have misidentified tip as the most critical subregion for conceptual processing either because tip and VL are functionally indistinct despite their cytoarchitectural differences or because, while tip and VL do indeed subserve distinct purposes, they mislocated the boundary between them. While both VL and tip had many task-responsive leads with similar response strengths and timing, Tiesinga et al. provided evidence of a functional distinction between these areas. Specifically, the temporal leads providing input to the tip and VL were clearly segregated along a dorso–ventral axis with VL receiving the majority of input from ventral PTC (vPTC) visual regions (FFA and lingual gyrus) and tip receiving more dorsal PTC (dPTC) input, which occurred with a longer delay (see Figure 3A). In addition, the feedback connections within TP were much stronger in tip than VL (Figure 2E). However, since subregion definition involved warping a group-level template onto individual brains, the border between VL and tip may have been mislocated. Thus, the critical region could be the most anterior portion of VL, yet its effects may have been mislabelled as tip. To assess the likelihood of this possibility, we performed an additional analysis based on Tiesinga et al.’s data. We first identified single electrodes that crossed from VL into tip in two patients. If Tiesinga et al.’s results were caused by mislocalising the boundary, only measurements of tip activity near the border would show these effects, while more anterior locations would not. This was not the case: both patients demonstrated strong responses and backward intra-TP connectivity throughout tip, which decreased around the border with VL, resulting in significant differences between the tip and VL locations on both metrics (Figure 2H). Thus, these data show a functional distinction along the longitudinal axis of the TP. Whether these differences correspond to smooth graded changes in function or a sharp boundary, they support the functional distinction between tip and VL, implicating tip as a more likely site for multimodal convergence than VL.

A second possibility is the social nature of the categories discriminated in Tiesinga et al. (2023). Some have proposed that TP is predominantly involved in processing social categories, particularly on the right (e.g., Olson et al., 2007, 2013; Ross & Olson, 2010; Wang et al., 2017; Zahn et al., 2007), perhaps due to the input from a valence spoke in OFC (Lambon Ralph et al., 2017; Rice et al., 2015, 2018). However, while the precise area identified across different fMRI comparisons of social and nonsocial semantics varies dramatically, it appears more frequently located in dorsal TP than in tip (Binney et al., 2016; Binney & Ramsey, 2020; Rice et al., 2018; Zahn et al., 2007), and the proposed anterior temporal face patch is focused on the basal surface and not the pole (Rajimehr et al., 2009). Indeed, using their specialised ECoG array, Abel et al. (2014) only identified stronger responses to people than landmarks or tools in leads focused in left and posterior ATL regions, with no such leads in right tip. Thus, both tip and VL regions of the left and right ATL appear to represent both social and nonsocial knowledge, and the social nature of the task is unlikely to explain the discrepancy with fMRI studies (Binney & Ramsey, 2020).

A more likely explanation relates to the limitations of fMRI and the nature of semantic representations. As the temporal resolution of fMRI is poor (∼3 s), it may simply miss the short-lived TP responses Tiesinga et al. (2023) have described. Semantic representations are highly distributed and the contribution of a particular location varies over time. As a result, activity at the same ATL electrode can change strength and even sign as a representation unfolds across a trial (Rogers et al., 2021). Poor temporal (or spatial) resolution means summarising over these changing positive and negative values and therefore potentially missing task critical regions. This is why we require precision measurements with high spatial and temporal resolution, such as Tiesinga et al.’s sEEG recordings. These issues are likely true of both tip and VL to some extent, yet, crucially these factors are likely to affect tip more if it is indeed the multimodal convergence point. The variability in a region’s contribution over time increases from sensory to multimodal conceptual areas and was identified in ATL but not PTC (Rogers et al., 2021). Increased temporal variability results in a lower likelihood of identifying a strong differential response, resulting in the observation that stronger responses tend to be identified earlier in processing streams. If this temporal variability continues to increase as we move further from sensory input, it would be highest at the multimodal convergence point. Indeed, plotting the amount of variance (Rogers et al., 2021) found in each electrode by its *y* coordinate (Figure 2G), we can see that the variance does appear to increase in a posterior to anterior direction, even within TP, reaching the highest value in the single tip electrode. Thus, the effects in tip may be missed in fMRI, or these effects may be identified alongside stronger activation (and therefore a peak) in VL, detracting attention from them. This possibility is supported by the apparent presence of tip activity within many of the fMRI studies that simply focus their attention on the vATL peaks (Binney et al., 2010; Jackson et al., 2015; Rice et al., 2018; Spitsyna et al., 2006; Visser & Lambon Ralph, 2011). The greater strength of the peaks in VL tends to be a measure of the larger or more consistent engagement of this region compared to baseline, not of how multimodal the response is. Combined with the observation that more peripheral areas have more consistent responses, fMRI peaks would be most likely to occur in the most peripheral area that overlaps between sensory modalities, not the most central. Overall, these dynamic changes being maximal in tip may be the most likely explanation for the discrepancy between the fMRI peaks and the sEEG results, as crucially many fMRI studies find activity spanning both VL and tip. Additionally, their impact may be exacerbated by the smoothing effects of fMRI, the focus on the precise location of a numerical peak, and an overreliance on visual stimuli (which may lead to more frequent identification of VL than the ATL areas that form part of other modalities’ processing streams). Further intracortical experiments comparing the engagement and connectivity of tip and VL in a range of semantic tasks presented across different modalities will be critical to adjudicate this discrepancy of fMRI peaks with spatiotemporally precise measurements, which so far were collected only with a simple visual semantic task.

## IMPORTANCE OF FEEDBACK AND SHORTCUT CONNECTIONS

To better emulate brain connectivity, Jackson et al. (2021) extended prior feedforward-only assessments (Rogers & McClelland, 2004) to assess the impact of semantic network architecture within a recurrent framework. Feedback connections were expected to be crucial to capture the complex dynamics of the semantic system, whereby activity patterns unfold over the full time course of conceptual access as they allow regions to continually influence each other (Clarke & Tyler, 2015; Kietzmann et al., 2019; Rogers et al., 2021; von Seth et al., 2023). Indeed, deep visual ANNs are better able to fit time-resolved neural data when they include both feedforward and feedback connections (Güçlü & van Gerven, 2017; Kietzmann et al., 2019; Nayebi et al., 2022; Thorat et al., 2022; von Seth et al., 2023). Both the reverse-engineered model and time-resolved investigations of the cortical semantic network demonstrate a high degree of interaction between regions at different levels in the processing stream. For example, MEG studies demonstrate recurrent connectivity between the ATL and vPTC during semantic task performance (Clarke et al., 2011; Rahimi et al., 2022), a pattern also identified by Tiesinga et al. (2023; involving tip more frequently than VL). However, with the greater spatial resolution afforded by sEEG, Tiesinga et al. were also able to demonstrate the importance and timings of both feedforward and feedback connections between VL and tip.

Tiesinga et al. (2023) found the latency of TP responses and connections varied considerably across patients, ranging from 125 to 225 ms (consistent with earlier studies; Chen et al., 2016; Clarke et al., 2011; Rahimi et al., 2023; Rogers et al., 2021; von Seth et al., 2023) and lasting approximately 100 ms. However, within patients these effects followed a stereotyped sequence, starting with feedforward connections from VL to tip, on average 140 ms after stimulus onset and 40 ms (20–70 ms across patients) before the TP responses (Figure 2D). This forward connection was quickly followed by a recurrent interaction, starting on average 12 ms (range 10–13 ms) before the TP responses. Remarkably, the feedback connections were stronger than the feedforward connections (both between tip and VL and within tip; Figure 2E), demonstrating the importance of feedback within the ATL. While the specific timings may be affected by the simple nature of the task employed, finding evidence of feedback connections at both inter- and intraregional levels supports the importance of the recurrent semantic processing simulated by Jackson et al. (2021). The spatial proximity of input and output regions in the reverse-engineered model precludes a detailed comparison of the timing of feedforward versus feedback connections. However, the simulation does result in a very short initial feedforward-only period while the unimodal sensory information propagates through the network to activate the multimodal hub, followed by persistent recurrent connectivity, consistent with the findings of Tiesinga et al.

**Figure 4.**
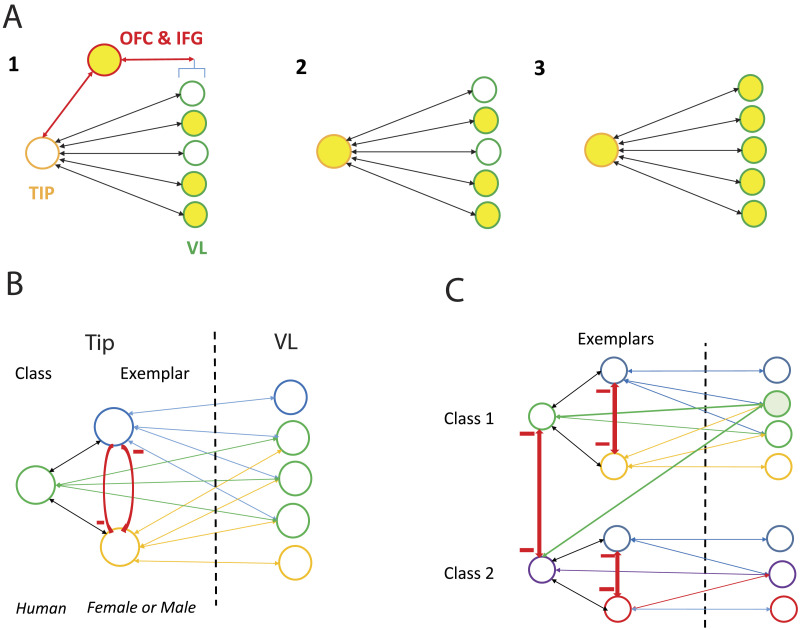
A putative scheme of how concepts are represented within TP, based on Tiesinga et al. (2023). (A) Concepts are represented by the combination of an abstract label in tip and unimodal features in other TP subregions, including visual features in VL. The three panels are a schematic representation of the three-step sequence of activation of the gender concept representation proposed by Tiesinga et al. Orange and green circles = neural populations in tip and VL, respectively; red circle = control regions; yellow filled circles = active populations. Note that each small circle represents many neurons and that while tip is proposed to link features, this is much more than a simple linear combination as, for example, nonlinear effects can be created by presynaptic inhibition. (B–C) Extension of the label–features scheme of Tiesinga et al. to two levels of categories: ordinate (classes) and exemplar levels. The semantic hierarchy arises from connections between label populations in tip. (B) Interaction between two exemplars of the same class (e.g., male and female humans). (C) Interactions between classes (e.g., humans and apes). All connections are proposed to be excitatory except those in red, which are inhibitory links between label populations at the same hierarchical level.

*How does this recurrent connectivity support semantic cognition?* Visual ANNs demonstrate that feedforward processing alone is sufficient for accurate object identification, particularly in relatively easy tasks, such as the gender discrimination employed by Tiesinga et al. (2023). However, this may not provide the efficiency needed to complete hard semantic tasks, quickly, with limited resources (Nayebi et al., 2022). Tiesinga et al. proposed that the feedback from the label in tip allowed pattern completion, activating features of the concept across the shallower TP regions. Hence, the complete representation of a concept would involve both tip and VL (see Figure 4 in Benefits of a Multilevel Representation of Concepts, below). Indeed, in the reverse-engineered model, the early activation of the multimodal hub allows the system to start to represent related sensory features across other senses and for the multimodal conceptual structure in the deep hub to influence processing in, and information transmission from, shallower sensory layers. The initial feedforward sweep may be sufficient to provide a coarse estimate of what an object might be, even when recognition is difficult. Employing multimodal information in a top-down manner at this stage could allow further processing to target specific diagnostic features promoting efficient recognition of objects, words, and people (Clarke & Tyler, 2015; Kveraga et al., 2007).

The reverse-engineered model demonstrated the importance of shortcut connections in semantic cognition for the first time. The addition of direct connections between shallow sensory and deep multimodal regions had a dramatic positive effect on both representation quality and speed of learning, despite the potential competition between fulfilling these two metrics. Moreover, these improvements were seen despite the shortcut connections representing a very small proportion of the total connections between sensory areas and the hub: there were around nine times as many connections at each step in the indirect route as there were shortcut connections.) While different connectivity proportions were examined, a level of sparsity is necessary to maintain biological plausibility; long-range white matter connections are present in the brain, yet sparse due to metabolic and packing constraints (Nelson & Bower, 1990; Plaut, 2002). While evidence for shortcut connections within the cortical semantic network is limited, the idea of multiple processing routes is highly compatible with the existence of two broadly distinguishable sets of white matter supporting interareal connectivity; the occipitotemporal projection system of U-fibres connecting neighbouring areas and the inferior longitudinal fasciculus bridging across longer distances (Bajada et al., 2017; Catani et al., 2003; Herbet et al., 2018). Moreover, tractography assessments demonstrate greater ATL connectivity with more distal posterior fusiform than mid-fusiform cortex (Bouhali et al., 2014). Similarly, connections are identified in nonhuman primates between higher order visual areas and cortical areas two levels further down in the hierarchy, for example, anterior dorsal TE receiving mainly from posterior dorsal TE supplemented by a weak projection from dorsal TEO (Kravitz et al., 2013). However, these structural connectivity assessments provide no information about whether and how these routes are utilised for semantic cognition. Nor can they tell us the direction of these possible shortcut connections.

By assessing the directed, effective connectivity of the ATL with high spatial and temporal precision, Tiesinga et al. (2023) demonstrated support for the involvement of both a multistep route and shortcut connections in semantic cognition. GC analyses identified 21 direct connections in the forward direction from vPTC to TP leads: 16 into VL and five directly projecting into tip (Supplementary Tables 10 and 11 in Tiesinga et al.). At least two of these five connections operated at 4 ms (Figure 3A). In the feedback direction, there were 20 direct connections from TP to vPTC leads, with 15 of these arising in the tip. At least five operated at 4 ms. Shortcut connections in the feedback direction therefore appear numerous, although it should be noted that there were more tip than VL leads (24 vs. 18) and all VL leads received feedback from tip, suggesting that many more connections supported feedback via a multistep route as well. Despite these factors, the number of feedback shortcut connections may be surprising given the need to have sparse structural connections over long distances due to packing and metabolic constraints. It may be that the long-distance structural connections are sparse, yet interactions in the feedback direction heavily depend upon this route. Alternatively, it may be that the number of shortcut connections identified by Tiesinga et al. was artificially inflated somewhat due to the inclusion of indirect connections which were estimated to be direct over a relatively long lag time. Connections at the 16 ms delay may reflect weak direct connections involving thin fibers or may be an incorrect characterisation of multiple rapid indirect connections. Overall, Tiesinga et al. provide initial novel evidence of sparse shortcut connections supporting bidirectional interactions between vPTC and tip (bypassing VL; Figure 1B, bottom). It is important to note that evidence for these shortcut connections were derived from Granger causality computations. GC is an inferential technique, with the direct connections identified open to alternative interpretations, such as very rapid multisynaptic pathways, particularly with the longer 16 ms delay. However, even when restricting the analyses to the 4 ms lag only, there was evidence of sparse shortcut connections. Future investigations should utilise additional methods to determine whether there is converging evidence of shortcut connections into the ATL and provide better estimations of their sparsity.

*Why should there be shortcut connections between vPTC and tip?* Jackson et al. (2021) found shortcut connections were effective at helping ameliorate the slowing caused by increasing depth. Error-driven learning across many layers is initially slow as it requires the manipulation of multiple connection weights (He et al., 2016; Saxe et al., 2019), which shortcut connections avoid by creating an alternative path through fewer layers. However, this does not explain why these connections also improved the quality of representations. Perhaps, quickly projecting coarse sensory information into the multimodal hub, activates conceptual representations able to support top-down semantic processing. This conceptual information could then impact sensory processing quickly by utilising the feedback shortcut connections from the tip to vPTC. Thus, shortcut connections may improve representations by encouraging dynamic interactions between perceptual and conceptual regions. These possibilities are highly compatible with the sEEG data, which showed relatively early responses in the TP and even earlier intra-TP connectivity, which Tiesinga et al. (2023) speculates to be dependent on responses below the detection threshold. Further investigation of shortcut connections will likely prove crucial to a mechanistic understanding of the dynamics of the cortical semantic system.

## INTERACTION BETWEEN CONTROL AND REPRESENTATION REGIONS

By varying connectivity in silico, Jackson et al. (2021) demonstrated that information signalling task-context should be received in more peripheral regions of the semantic network and not the core multimodal region. Shielding the multimodal hub from the direct effects of task context is imperative to allow it to extract the full conceptual structure based on the relationship between features presented across different contexts and episodes. To date, the functional connectivity of semantic control and representation regions has been assessed with fMRI (Jackson et al., 2016; Jung & Lambon Ralph, 2023), which has a notoriously poor temporal resolution, and MEG (Farahibozorg et al., 2022; Rahimi et al., 2022, 2023), which has a limited spatial resolution. Thus, Jackson et al. provided a clear prediction as to how semantic representation and control regions interact (Figure 1A, bottom), yet existing studies provide little evidence to adjudicate this hypothesis. With the excellent spatiotemporal resolution of sEEG, Tiesinga et al. (2023) were able to provide unique evidence illuminating how and where frontal regions interact with subregions of the ATL hub during basic semantic processing. Specifically, the strength and timing of the feedforward connection from vPTC to VL was mediated by the OFC. By controlling the timing of these inputs, OFC indirectly determined the duration of the TP responses. Furthermore, within the TP, the feedforward connection from VL to tip was mediated by both OFC and inferior frontal gyrus (IFG; Figure 3B). Both findings are consistent with Jackson et al.’s prediction that control regions should not act directly at the multimodal convergence point but instead influence more peripheral areas (Figure 1). Moreover, Tiesinga et al. specifically showed the influence of these potential control areas on the early feedforward input from unimodal sensory streams and not the strong multimodal feedback signal. Further investigations will be crucial to assess the interaction between control and representation regions in more controlled, demanding semantic tasks. In particular, recording of the left hemisphere will allow a better assessment of the role of semantic control regions, including the IFG which demonstrates relatively left-lateralised activation (Jackson, 2021). The interaction between the frontal cortex and the ATL has previously been hypothesised to occur via posterior temporal control regions (Davey et al., 2015; Hodgson et al., 2023; Jackson, 2021), based on their connectivity (Catani et al., 2005; Jackson et al., 2016; Matsumoto et al., 2004) and the apparent functional similarity of IFG and PTC (Jefferies, 2013; Thompson et al., 2015). Tiesinga et al. found little evidence for this hypothesis. However, critically, these assessments were performed only in the right hemisphere, while only the left PTC appears important for semantic control (Davey et al., 2015; Jackson, 2021; Jefferies, 2013; Jefferies & Lambon Ralph, 2006; Noonan et al., 2013; Thompson et al., 2015). Future intracortical studies should determine whether this is an additional important route in the left hemisphere.

*How do these frontotemporal connections support controlled semantic cognition?* Semantic control assessments typically focus on the ability to selectively retrieve task-relevant information, as modelled in Jackson et al. (2021). Frontal areas may shape the information retrieved within the hub by controlling its inputs, manipulating the interaction between the hub and its spokes in a task-appropriate manner. Activity in the hub would then affect responses in earlier areas via its strong feedback connections, explaining the effects of task context in sensory areas (e.g., Martin et al., 1995; as simulated in Jackson et al.). The dynamic interaction between control regions, the multimodal hub and sensory regions may collectively shape ongoing retrieval, informed by both the information accessed so far and the task context. While the specific interactions would differ between different tasks, a high degree of interaction would support efficient, task-relevant processing. For example, when given the task to decide if an object is dangerous, determining that it is a plant would allow further processing to focus on different features (e.g., toxicity, colour) than if it were an animal (e.g., size, sharp teeth). Similarly, when determining whether two words are related, we must access features of the first word and use these to guide our search for relevant features of the second word. Intriguingly, most research on semantic control has focused on the IFG as the critical frontal area, although meta-analyses (Figure 3C) show consistent activation across a broad region, including lateral OFC (Binder & Desai, 2011; Jackson et al., 2021; Noonan et al., 2013), which has strong structural connections with the ATL (Von Der Heide et al., 2013). In contrast, Tiesinga et al. (2023) highlighted a greater role for OFC, which includes most of the electrodes mediating intra-TP connectivity (Figure 3B), and all those responsible for controlling the timing of the inputs to TP. The differing spatial focus of the two methods could be explained, at least in part, by poor fMRI signal within OFC or by the focus of the intracortical investigation on the right hemisphere, as right IFG appears less important for the control of meaningful stimuli (Hodgson et al., 2023; Jackson et al., 2021; Jefferies, 2013; Thompson et al., 2016). Indeed, comparison of these regions across the two hemispheres will be crucial, and Tiesinga et al.’s analyses focusing on the right hemisphere should not be considered evidence against the crucial role of the left IFG in semantic control. Moreover, the importance of the OFC in Tiesinga et al.’s analyses, may not be a general semantic control effect. For example, the social nature of the gender task may drive the OFC response. Within the graded hub-and-spoke view, OFC has predominantly been considered a spoke for emotional content or valence, providing input to the ATL via the rostral TP (Patterson et al., 2007; Rice et al., 2018). This is consistent with the direct input from OFC to VL identified by Tiesinga et al., but less so with the initial direction of effective connectivity being from VL to tip.

Alternatively, the IFG and OFC may underpin distinct control-related processes, consistent with their differing functional and structural connectivity patterns; the OFC is structurally connected to TP via the uncinate fasciculus, whereas IFG connects to PTC control areas via the arcuate fasciculus (Catani et al., 2005). IFG may be central for semantic control as typically assessed with tasks requiring the manipulation of semantic knowledge to resolve ambiguity, inhibit dominant features and select less dominant features as required, yet show limited involvement in Tiesinga et al. (2023) simply as the semantic control demands were low in the gender identification task, which was relatively easy (reaction times were around 820 ms) and did not emphasise these key semantic control processes. In contrast, the OFC involvement may not reflect semantic control as typically conceived but rather a related, yet separable process. For example, OFC has previously been associated with the top-down support of visual object recognition, providing contextual support to help identify an object efficiently based on the statistical probabilities of its environment (Kveraga et al., 2007). Alternatively, Tiesinga et al.’s analyses may lead us to consider an alternative hypothesis that OFC may assess whether an appropriate amount of evidence has accumulated to make the specific judgement required by the task. This would allow response times to differ between tasks varying in the required depth of semantic processing (e.g., “Is it a bird?” vs. “Is it a robin?”) by curtailing unnecessary processing within TP once the relevant behavioural output is possible. Manipulating this threshold allows an accurate, yet fast response to any given task. Tiesinga et al. demonstrated that OFC modulated the duration of the input to VL from vPTC, which determined the timing of the TP responses, perhaps allowing them to be very brief for the easy gender task. Within the conceptualisation by Tiesinga et al., this would mean OFC modulates the input to the tip neurons representing the gender concept labels, to determine how stringently it should test the evidence provided by the semantic features collected by VL and identify whether sufficient evidence has accumulated. Alternatively, the role of OFC in reward processing (Balleine et al., 2011; Rolls, 2023; Rudebeck & Murray, 2011; Walton et al., 2011) could point to a broader role in driving semantic processing to favour rewarding—that is, task-relevant or socially significant—features. Currently, the functional significance of the OFC in semantic control is unknown and there are multiple ways to interpret Tiesinga et al.’s findings. However, the apparent control OFC exerts over TP responses is an intriguing avenue for future exploration. Further intracortical investigations of executively demanding semantic tasks are necessary to distinguish the possible roles of left and right OFC and IFG in controlled semantic cognition.

## BENEFITS OF A MULTILEVEL REPRESENTATION OF CONCEPTS

From their pioneering recordings, Tiesinga et al. (2023) hypothesised a two-level representation of concrete concepts in TP with only the multimodal concept label stored in tip, and the defining unimodal, sensory features stored in other TP subregions, including VL for vision. This organisation allows many concepts to be represented in a small space, as many labels can be stored in tip and the same unimodal features can subserve multiple concepts. Such efficient storage is crucial given the many concepts humans can acquire. Moreover, the activation of shared features allows generalisation between similar concepts. Importantly, these advantages depend on the conceptual representations being distributed across many neurons, as assumed by both Tiesinga et al. and the Jackson et al. (2021) model. Indeed, Tiesinga et al. demonstrated that each exemplar activates about two thirds of all tip neurons. This distributed processing stands in contrast to the sparse coding in hippocampus (Quiroga, 2012), consistent with the general view that episodic and semantic memory should use sparse and distributed coding, respectively (Frankland & Bontempi, 2005; McClelland et al., 1995).

Tiesinga et al. (2023) derived the two-level representation by distinguishing three steps of TP activity when matching a visual item to a concept (Figure 4A): (1) VL collects the evidence in favour of a concept (here either male or female); (2) when this evidence is sufficient for one particular exemplar, as determined by PFC, tip is activated; (3) this tip activation automatically feeds back to VL and the other unimodal parts of TP, such as the dorsal area for audition, without outside control. Thus, the feedback from tip results in a pattern completion process, whereby all the concepts’ features are activated, even those that were not initially activated by the presentation of the item. This is possible only owing to the convergence of the different sensory streams at one location (i.e., a multimodal convergence point or hub) in a network with multiple layers (i.e., depth), and, as Jackson et al. demonstrates, this results in gradual changes in the extent to which areas are multimodal as the feedback provides multimodal influences on earlier regions. Thus, performing pattern completion across modalities requires a graded deep hub-and-spoke architecture.

The two-level representation, proposed by Tiesinga et al. (2023), could also allow the implementation of a hierarchical structure of categories (Figure 4B and C; Quillian, 1968; Warrington, 1975) whereby tip includes both class and exemplar labels. Neuronal tip populations acting as labels of two exemplars sit at the same level (here male and female) and may be linked by reciprocal inhibitory connections, along with populations representing labels of the same class level. On the other hand, representations of the higher class level (humans) may be based on the overlap of features activated in the lower exemplar level (in this case, the features associated with both male and female humans) resulting in both direct (excitatory) connections between the class label and these features, and indirect connections via the exemplar labels.

While this extended Tiesinga et al. (2023) two-level scheme is reminiscent of Quillian’s hierarchical propositional model of living things (Quillian, 1968), semantic knowledge is accessible at multiple levels of the hierarchy and not just the superordinate level, as labels at different hierarchical levels in tip are linked to features in VL. Furthermore, given its similarity to recurrent neural networks of the hub-and-spoke theory (Jackson et al., 2021; Rogers & McClelland, 2004), the extended Tiesinga et al. scheme does not suffer from the drawbacks of hierarchical propositional models (McClelland & Rogers, 2003). First, both class and exemplar labels are linked directly to their semantic features, leading to equally fast verification (McCloskey & Glucksberg, 1979; Murphy & Brownell, 1985), while the stronger link between class labels and their properties may reflect the combination of their direct links and indirect links (via the exemplar level; Figure 4C). Second, Step 2 in the Tiesinga et al. scheme is reminiscent of category verification in ANNs, which is based on similarity between representations of items and categories, a more efficient strategy than searching a hierarchical tree (McClelland & Rogers, 2003). Finally, the extended Tiesinga et al. scheme predicts an increasing duration of ATL activity as tasks address higher levels of the hierarchy. Indeed, the indirect links between label and features for superordinate classes (e.g., living things vs. artefacts) would involve a greater number of synapses than the connections between features and subordinate exemplar labels (e.g., female, male), allowing multiple recurrent loops using the feedback connections within tip and between tip and VL. Consistent with this prediction, the TP activation for superordinate level animacy judgements reported by Rogers et al. (2021) was much longer than those for the exemplar-level gender judgements in Tiesinga et al. However, because higher level categories also have direct label–features connections (Figure 4C), tasks involving low and high hierarchical levels elicited equally fast EEG responses (Thorpe et al., 1996).

Thus, it seems that the small modification to the Tiesinga et al. (2023) scheme for representing concepts in TP, introduced here, allows us to reconcile parallel distributed and hierarchical semantic processing. The modified scheme also reconciles category- and feature-based views of semantic representation (Frisby et al., 2023), and could even accomodate vector space views, as the activity in VL could represent noninterpretable dimensions as much as sensorimotor features, and activity in tip may reflect the point in vector space defined by these dimensions. Furthermore, in this scheme semantic representation is grounded (Frisby et al., 2023), as activity in VL feeds back to the visual and other sensorimotor areas that support its feature/dimension representation. However, because it exhibits pattern completion it also has a self-contained element.

In addition to providing insights into the semantic representations, the two-level concept representation in TP may have wider implications. Indeed, the functional connectivity between tip and superior posterior temporal regions of the language network (Fedorenko et al., 2024; Hickok & Poeppel, 2007; Price, 2012; Price et al., 2005) may allow one-to-one links between the concept labels and the corresponding verbal labels. Combined with those between the hub and the spokes, these links may explain the wide distribution of linguistic meaning across cortex, both within and outside the language network (Pereira et al., 2018; Wang et al., 2018).

## IMPLICATIONS

In this article, we highlight the remarkable convergence between recent computational modelling experiments and precision physiological recordings of the semantic system (Figure 1). A key reason for this success is the high spatial and, in particular, temporal resolution of the sEEG technique used by Tiesinga et al. (2023). Indeed, the recorded ATL responses would likely go undetected in fMRI due to both their extremely short duration and the dynamic nature of the code utilised by the distributed concept representations (Rogers et al., 2021). Of course, the spatial specificity could be improved even further with single cell recordings (Leonard et al., 2024; Xie et al., 2024). However, these have yet to be performed in the ATL. A second crucial factor is the reverse-engineering approach Jackson et al. (2021) adopted. While a single model could be more or less suited to a function, systematic comparison across models allows the identification of beneficial architectural features. However, the reverse-engineered model and the brain could improve performance in distinct ways. Indeed, recent advances in the performance of deep visual ANNs have actually reduced their similarity to the cortical visual system (Schrimpf et al., 2020). This discrepancy is likely a function of the models’ increasing complexity, with higher performing models including far more units and connections, a solution that is not available to the biologically constrained brain. Jackson et al. circumvented this issue by matching model complexity. The convergence between the cortical data and the optimal model provides a crucial validation of the reverse-engineering approach’s ability to identify brainlike solutions. This occurred despite Tiesinga et al.’s use of a simple semantic task and focus on the lesser observed right hemisphere. Consequently, additional investigations employing precision recording will be crucial to validate and extend our knowledge of these points of convergence. Most intriguingly, Tiesinga et al.’s results provide initial evidence supporting a novel prediction generated by the reverse-engineered model of controlled semantic cognition: the importance of utilising shortcut connections between distal layers. Shortcut connections may be critical to accurately simulate the complex dynamics of the semantic system in future neuroanatomically inspired computational models. Indeed, more neurobiologically plausible patterns of recurrent connectivity and sparse long-range connections may improve the ability of deep ANNs to provide brainlike simulations of a multitude of cognitive processes. Furthermore, if shortcut connections allow multimodal regions to impact distal sensory areas with top-down conceptual knowledge, all state-of-the-art deep ANNs performing visual or auditory identification based on a single sensory modality in isolation are inherently limited.

Tiesinga et al.’s (2023) sEEG recordings represent the end of a long wait for data pertaining to the operations within the semantic hub, allowing comparison of the Jackson et al. (2021) modelling to the brain at a more granular level. Together the neural and computational assessments highlighted the functional heterogeneity of cytoarchitectonically distinct ATL subregions, including the importance of tip and VL. Simulating multiple subregions at different processing depths within the ATL provides a mechanistic explanation of the graded changes in the modality of information represented across this hub. Tiesinga et al. aligned the deepest model layer with tip, while previous work has typically considered VL to be the multimodal convergence point. Determining which subregion sits atop this organisational hierarchy and is therefore the centre of the graded hub, will be critical to understand the functional organisation of the ATL and inform neuroanatomically precise models of semantic cognition in health and disorder. Tip and VL appear to have complementary roles, with Tiesinga et al. speculating that VL represents the higher order visual features present in the input and that tip represents the multimodal combination of features defining a concept (the concept label; see Benefits of a Multilevel Representation of Concepts). They hypothesised that VL must provide adequate evidence of a particular concept to activate tip, which then engages additional sensory features in the other shallower unimodal regions. Jackson et al. demonstrated how this process of pattern completion across different spokes may be performed in a context-dependent manner. Indeed, aligning the neural and computational evidence illuminated how prefrontal control regions impact ATL responses. Surprisingly, this revealed a potential additional role of the OFC in semantic control that may have been obscured by the prior focus on nearby IFG. This possible involvement in warping the semantic space to fit the task context is consistent with recent suggestions that OFC represents the task state space (Liao et al., 2024; Schuck et al., 2016; Wilson et al., 2014). Alternatively, OFC may have a related but distinct role, for example, in determining when sufficient semantic processing has occurred. The relative contribution of the OFC and IFG in a more controlled, demanding semantic task remains to be seen. OFC controlled both the timing of the input to VL and the connection from VL to tip (alongside IFG). As predicted by the reverse-engineered model, this PFC mediation did not directly impact the area proposed to be the multimodal convergence point.

By bridging the computational and neural literature on the ATL hub, this perspective has generated greater understanding of how the brain can meet the core yet conflicting, requirements of controlled semantic cognition. Now is a crucial time to gain greater understanding of how this is made possible by the architecture and mechanics of the human cortical system, as generative AI models, such as ChatGPT, based on large language models are having a disruptive impact on society (Conroy, 2023; Hutson, 2021a, 2021b). These models have the semblance of semantic cognition skills, appearing to store conceptual knowledge and use it flexibly as the context requires, using a vector-based representation similar to those proposed earlier for the semantic system (Piantadosi et al., 2024). Yet they do not understand meanings per se, instead simply repeating previously used phrases that seem relevant for the context, known as “parroting” (Bender et al., 2021; Fayyad, 2023; Mitchell & Krakauer, 2023). Moreover they require a vast amount of training and resources to learn (Bartoldson et al., 2023; Luccioni et al., 2023). In contrast, the human brain is capable of a much more remarkable feat of extracting concepts from multiple events, each containing only a subset of the important features, while also producing only the subset of features relevant to the current task demands. Moreover, it meets these core, yet conflicting, requirements of controlled semantic cognition with far fewer resources and training. Understanding how this is possible is crucial to advance both our understanding of the brain and the development of new generations of AI technology.

## CONCLUSIONS AND FUTURE DIRECTIONS

Within this perspective, we demonstrate how we can advance our understanding of the neural architecture for controlled semantic cognition by combining evidence from in silico experiments and in vivo precision measurements. These approaches are symbiotic; computational models must be encouraged to be brainlike and neural measures require formal, mechanistic explanation (Doerig et al., 2023). Thus, we demonstrate the power of computational cognitive neuroscience to bridge the explanatory gap between brain and behaviour. Only through continual translation between computational modelling and neuronal observations are we able to refine and test precise, mechanistic theories of how complex functions arise from the brain.

However, the process of applying this to understand controlled semantic cognition has only just begun. Indeed, the convergence identified here has only been demonstrated within the right hemisphere, for unimodal visual stimuli of a single type with a very simple semantic task. Confronting the model with more sEEG (Mercier et al., 2022; Rogers et al., 2021; Zhu et al., 2022) and single cell recordings (Bausch et al., 2021; Jamali et al., 2021, 2024; Khanna et al., 2024; Paulk et al., 2022; Xie et al., 2024; Zheng et al., 2022) across hemispheres and varied semantic tasks remains an important task for the future. Future experiments should: (1) directly compare the responses and connections of each cytoarchitectural region in the left and right ATL using sEEG; (2) present semantic stimuli across different modalities during sEEG recording to directly test which cytoarchitectural regions show multimodal responses across time, and (3) distinguish the connectivity patterns associated with semantic control versus representation processes and the roles of IFG and OFC by manipulating semantic control demands. In addition, neuroanatomically constrained models of controlled semantic cognition should be constructed to allow closer convergence with these cortical recordings, and their ability to simulate the dynamics of semantic processing across tasks tested.

## FUNDING INFORMATION

Paul Tiesinga, National Institute of Mental Health (https://dx.doi.org/10.13039/100000025), Award ID: R01MH123687.

## AUTHOR CONTRIBUTIONS

**Rebecca L. Jackson**: Conceptualization; Visualization; Writing – original draft; Writing – review & editing. **Guy A. Orban**: Conceptualization; Visualization; Writing – original draft; Writing – review & editing. **Paul Tiesinga**: Conceptualization; Visualization; Writing – original draft; Writing – review & editing.

## DATA AND CODE AVAILABILITY

No new data or code were produced for this manuscript.

## TECHNICAL TERMS

Semantic cognition: Learning and using concepts, that is, the meanings associated with words, living things, and objects.
Stereo electroencephalography (sEEG): Recording electrical signals from electrodes implanted within the brain.
Granger causality (GC): A method for assessing whether the values of one signal over time influence the values of another signal over time.
